# Endothelin A Receptor Antagonist, Atrasentan, Attenuates Renal and Cardiac Dysfunction in Dahl Salt-Hypertensive Rats in a Blood Pressure Independent Manner

**DOI:** 10.1371/journal.pone.0121664

**Published:** 2015-03-16

**Authors:** Mohammed A. Samad, Ui Kyoung Kim, Joshua J. Kang, Qingen Ke, Peter M. Kang

**Affiliations:** 1 Cardiovascular Institute, Beth Israel Deaconess Medical Center, Harvard Medical School, Boston, Massachusetts, United States of America; 2 Department of BIN Fusion Technology, Chonbuk National University, Jeonju, South Korea; University of Miami School of Medicine, UNITED STATES

## Abstract

Proteinuria is a hallmark of chronic kidney disease (CKD) and cardiovascular disease (CVD), and a good predictor of clinical outcome. Selective endothelin A (ET_A_) receptor antagonist used with renin-angiotensin system (RAS) inhibitors prevents development of proteinuria in CKD. However, whether the improvement in proteinuria would have beneficial effects on CVD, independent of RAS inhibition, is not well understood. In this study, we investigated whether atrasentan, an ET_A_ receptor antagonist, has renal and cardiovascular effects independent of RAS inhibition. Male Dahl salt sensitive (DSS) rats, at six weeks of age, received water with or without different doses of atrasentan and/or enalapril under high salt (HS) diet or normal diet (ND) for 6 weeks. At the end of 12th week, atrasentan at a moderate dose significantly attenuated proteinuria and serum creatinine without reducing mean arterial pressure (MAP), thereby preventing cardiac hypertrophy and improving cardiac function. ACE inhibitor enalapril at a dose that did not significantly lowered BP, attenuated cardiac hypertrophy while moderately improving cardiac function without reducing proteinuria and serum creatinine level. Nonetheless, combined therapy of atrasentan and enalapril that does not altering BP exerted additional cardioprotective effect. Based on these findings, we conclude that BP independent monotherapy of ET_A_ receptor antagonist attenuates the progression of CKD and significantly mitigates CVD independent of RAS inhibition.

## Introduction

CKD has been recognized as a worldwide public health issue, affecting 6% to 11% of the population in the developed world [1,2]. It is an independent risk factor for CVD and is associated with increased morbidity and mortality [3,4]. Over 80% of CKD patients at the initiation of hemodialysis suffer from left ventricular hypertrophy (LVH), an abnormality strongly linked with increased mortality risk [5,6]. In similar context, the rate of cardiovascular-related mortality in CKD patients is 10–20 times higher than in the general population [7]. The increased risk of CVD in CKD patients are mainly associated with not only the high prevalence of traditional risk factors, such as hypertension and diabetes [1], but also with non-traditional risk factors, such as albuminuria, renal insufficiency, structural and functional abnormalities of the heart [1,8].

Proteinuria is a hallmark of CKD, and also a major factor for the progression to CKD [9]. Recent clinical studies have further revealed that proteinuria is an independent risk factor for cardiovascular events, and a predictor of mortality prognosis [3,9]. Importantly, reduction of proteinuria is associated with the improvements of cardiovascular outcome in those patients with and without CKD [10,11]. Current treatments for proteinuria focus on blood pressure (BP) reduction [12], ideally using ACEi and angiotensin receptor blockers, both of which are thought to decrease proteinuria to a greater extent than accounted for by BP lowering alone [11–13]. Nevertheless, many CKD patients have significant residual proteinuria, despite of an optimal treatment [14]. Thus, there is a great need for complementary treatments which can effectively augment the reduction of the progressive loss of kidney function and proteinuria [15–17].

Endothelins (ETs) are endothelial cell–derived vasoconstrictor and vasopressor [18,19], and mediate their biological activities through the endothelin receptors A (ET_A_) and B (ET_B_). The binding of ET to ET_A_ receptor increases vasoconstriction and retention of sodium, subsequently leading to increased BP [20]. ET_A_ receptor antagonists have been shown to be effective in abrogating proteinuria and kidney fibrosis in various models of rats with kidney damage [21]. A recent Phase 2 dose-ranging study showed that atrasentan, a highly selective ET_A_ receptor antagonist, used in conjunction with RAS inhibitors, may reduce proteinuria and stall CKD progression in patients with diabetic nephropathy [22]. However, it has not been clearly identified whether improvement in proteinuria correlates with cardiovascular outcome, and whether the beneficial effects of atrasentan is independent to renin-angiotensin system (RAS) inhibition. In this study, with the Dahl salt sensitive (DSS) rat model, we examined the degree of proteinuria correlates with cardiovascular abnormalities, and tested our hypothesis that atrasentan, through inhibition of specific ET_A_ receptor, has beneficial renal and cardiovascular effects that are independent of RAS inhibition.

## Materials and Methods

### Materials

Materials and chemicals were obtained from following sources: 3-(4,5-dimethylthiazol-2-yl)-2,5-diphenyltetrazolium bromide (MTT) (Sigma-Aldrich, USA), Dimethyl sulfoxide (Sigma-Aldrich, USA), Enalapril (Sigma-Aldrich, USA) and IDEXX Catalyst Test kit for creatinine (IDEXX, USA). Atrasentan was provided by Abbott Labs, Abbott Park, IL.

### Animal model and experimental groups

Male DSS rats (Harlan Sprague–Dawley, Indianapolis, IN) were fed a normal diet until 6 weeks of age. To generate cardiac hypertrophy, animals were fed a high salt (HS) diet (6% NaCl) for the following 6 weeks as described previously [23,24]. To study the effects of atrasentan, rats fed HS diet were divided and treated for 6 weeks as follows: (i) HS + vehicle (V), (ii) HS + low dose atrasentan (2.5 mg/kg/day), (iii) HS + moderate dose atrasentan (5 mg/kg/day), (iv) HS + high dose atrasentan (10 mg/kg/day), (v) HS+ enalapril (10 mg/kg/day), (vi) HS + combined therapy of moderate dose atrasentan (5 mg/kg/day) and enalapril (10 mg/kg/day), in drinking water. Male and female have different baseline heart weights as well as different responses to the high salt diet. To avoid these gender-related variations, we used the same gender DSS rats for our study.

Atrasentan, selective ET_A_ blocker (around 1000 times greater affinity for ET_A_ vs ET_B_) provides maximum ET_A_ blocked and selectivity at the dose 5 mg/kg/day *in vivo* [25] and significantly attenuated proteinuria streptozotocin-induced rat model of diabetes [26]. Based on the previous studies, we defined 2.5, 5.0 and 10.0 mg/kg/day as low, moderate and high doses for this study. In baseline atrasentan dose study, we found that low dose doesn’t have significant effect on renal and cardiac system, and high dose has a significant effect on BP. Moderate dose of atrasentan has no significant effect on BP but significantly attenuates reno-cardiac dysfunction. To meet the main objective of present study, whether reno-cardiac beneficial effect of BP independent dose of atrasentan is independent of RAS inhibition, moderate dose of atrasentan was used in combination with enalapril. Rats fed normal diet were divided and treated for 6 weeks as follows: (i) normal diet (ND) + V, and (ii) ND + high dose atrasentan (10 mg/kg/day) in drinking water. Data were obtained at the end of 12 weeks from HS+V and ND+V treated group, respectively. The dose of atrasentan and enalapril were adjusted weekly according to actual body weight. At the end of 12 weeks, we examined morphometric measures, cardiac function using noninvasive and invasive physiologic methods, histopathology, and biochemical/molecular changes related to cardiac stress. All procedures were submitted to, approved by and performed in accordance with the Beth Israel Deaconess Medical Center Institutional Animal Care and Use Committee (IACUC) guidelines.

### Blood pressure measurement and echocardiography

Direct BP was measured with a 1.4 F micro-tip pressure catheter (model SPR-671, Millar Instruments, Inc. Houston, TX) inserting into the carotid artery under isoflurane anesthesia. The data was recorded and analyzed with computer software (PowerLab, Chart 5, ADInstruments, CO). Echocardiography was performed under isoflurane anesthesia as described previously [23] at the end of study period on each group of animal.

### Adult rat cardiomyocytes (ARCM) culture studies

ARCMs were isolated and cultured from 6-week-old female DSS rats by enzymatic dissociation using 0.3% collagenase, according to a previously published protocol [27,28]. To investigate the protective effects of atrasentan and enalapril against i*n vitro* cardiac hypertrophy, ARCM cells were pre-treated with or without various concentrations of atrasentan and enalapril 30 minutes prior to the treatment with hypertrophic stimuli phenylephrine (100 μM). Cells were harvested after 48hrs of treatment to determine cardiomyocyte hypertrophy.

### Biochemical analysis

Blood samples were collected at the end of study period from each group of animals. Then, serum samples were collected and serum creatinine levels were measured by IDEXX Catalyst Test kit for creatinine according to the manufacturer's instructions with Catalyst Dx Chemistry Analyzer (IDEXX, ME, USA). Urine samples were collected at the end of experiment and protein excretions were detected with Bradford protein assay.

### Reverse transcription polymerase chain reaction (RT-PCR) for mRNA expression

Heart tissues were collected and processed for molecular analysis at the end of 12 weeks. Semi-quantitative RT-PCR was performed as described previously [24,29]. Ribosomal 18S acted as internal controls, and all RT-PCR signals were normalized to 18S expression.

### Histological studies

Kidneys were fixed in 10% formalin and paraffin-embedded. Sections were stained with Masson-Trichrome (MT) at the Histology Core facility at Beth Israel Deaconess Medical Center. Tubulointerstitial injury was determined by evaluation of slides stained with MT to quantify the area of tissue fibrosis (blue) versus unstained region of image as described previously [24,29,30].

### Statistical analysis

Data were expressed as means ± SEM. Comparisons between and within groups were conducted with unpaired Student’s *t*-tests and repeated-measures one-way ANOVA (followed by Bonferroni: compare all pairs of columns) using GraphPad Prism 5.0 (San Diego, CA), respectively.

## Results

### Atrasentan attenuate phenylephrine induced hypertrophy in ARCM

**Fig 1:**
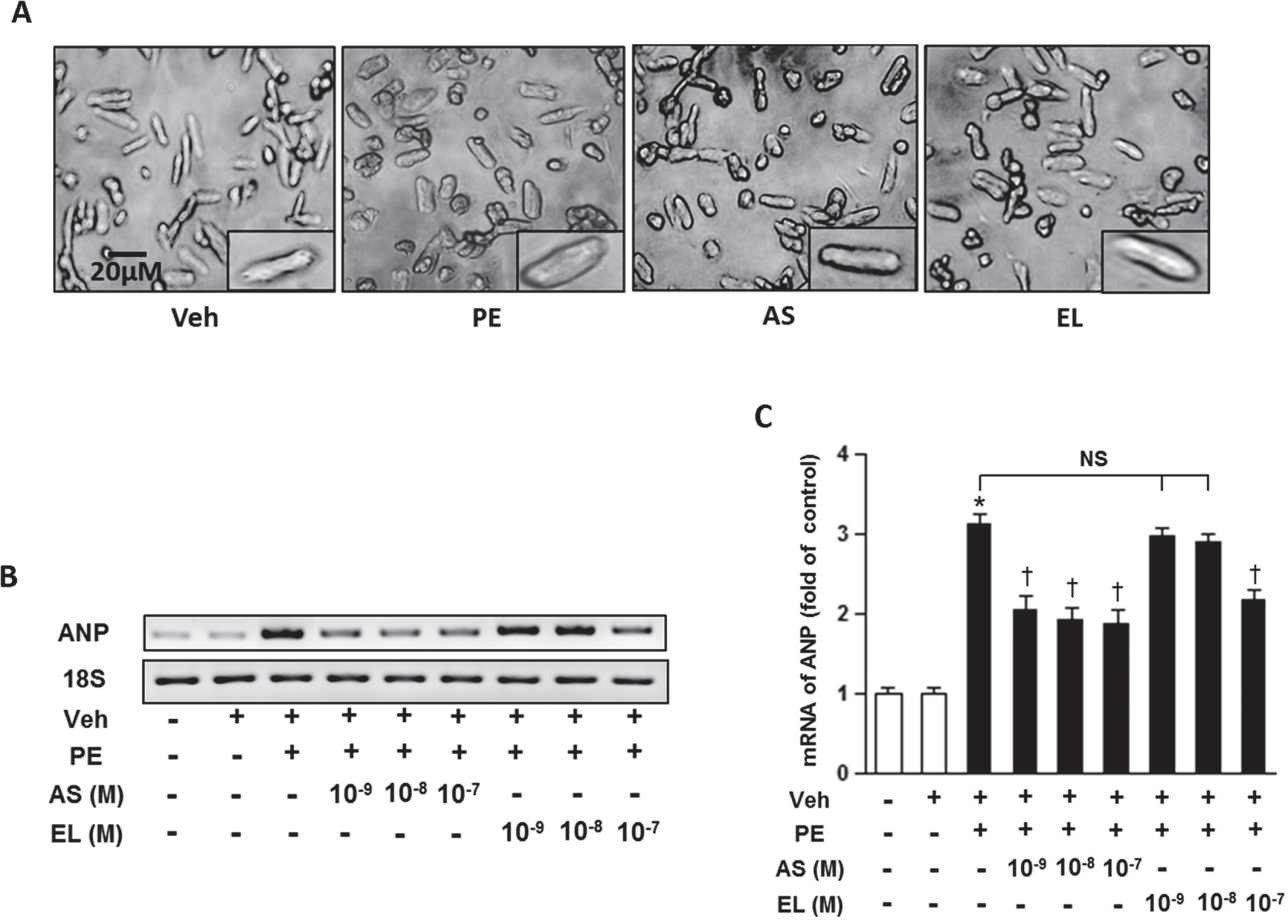
Effect of atrasentan on cardiac hypertrophy *in vitro*. *A*. Representative pictures of ARCM cultures. *B*. Representative mRNA expression of ANP. *C*. Quantitative analysis of mRNA expression of ANP. 18S expression is shown for internal loading control. The experiments performed in triplicate and results are expressed as means ± SEM. *n* = 3/group. *P < 0.05; *P < 0.05 vs. negative control group; ^†^ *P* < 0.05 vs. PE (100 nM) group. Veh, vehicle; PE, phenylephrine; AS, atrasentan; EL, enalapril.

We examined the anti-hypertrophic effect of atrasentan and enalapril in ARCMs by pre-treating the cells with various concentrations of atrasentan and enalapril 30 minutes prior to treatment with phenylephrine (100 μM), a well-known hypertrophic stimulus. After exposing the cells to phenylephrine with various doses of atrasentan and enalapril for 48hrs, the cells were collected and analyzed for evidence of hypertrophy. There were significant increase in cell size (Fig. 1A) and atrial natriuretic peptide (ANP) mRNA expression in phenylephrine treated group (Fig. 1 B-C). Atrasentan significantly reduced the phenylephrine induced activation of ANP mRNA expression compare to the vehicle treatment even at a very low concentration of 10^–9^ M. Nonetheless, only high dose (10^–7^ M) of enalapril significantly attenuated phenylephrine induced hypertrophy.

### Atrasentan effects on cardiac hypertrophy and dysfunction/heart failure

The DSS rat is a well-established animal model of high salt diet-induced cardiac hypertrophy and heart failure [23,27]. At 6 weeks of age, the male DSS rats received water with or without different dose of atrasentan and/or enalapril under HS diet or ND for 6 weeks. Organ weights were adjusted to the tibia length (TL) and body weight (BW). There were significant increases in heart weight (HW) as well as lung weight (LuW) in DSS rats receiving high salt diet with vehicle when compared with their normal diet littermates, which indicates that HS diet rats developed significant cardiac hypertrophy after 6 weeks of HS diet (Table 1).

**Table 1:** Morphometric analysis of DSS rats treated with atrasentan.

	Normal Diet		HS diet					
	Veh	AS 10 mg	Veh	AS 2.5 mg	AS 5 mg	AS 10 mg	EL 10mg	AS 5 mg + EL 10mg
HW/BW, mg/g	3.09±0.09	3.03±0.07	5.37±0.48*	4.81±0.30	4.09±0.25^†^	3.70±0.20^†^	4.39±0.27^†^	3.84±0.17^†^
LuW/BW, mg/g	3.86±0.08	3.73±0.07	6.54±1.51*	5.07±0.64	4.08±0.20^†^	3.88±0.10^†^	4.47±0.41^†^	3.93±0.11^†^
HW/TL, mg/mm	30.84±0.83	30.79±0.69	45.23±3.53*	43.16±3.32	36.23±1.50^†^	35.8±1.58^†^	36.89±0.83^†^	34.16±0.95^†^
LuW/TL, mg/mm	37.41±1.10	35.10±1.60	48.25±6.47*	43.32±5.49	37.48±1.29^†^	36.26±2.03^†^	37.98±2.24^†^	37.5±2.18^†^

The results are expressed as means ± SEM. *n* = 5–8 in each group.

*P < 0.05 vs. normal diet with vehicle treated group;

^†^ *P* < 0.05 vs. high salt (6%) diet with vehicle treated group.

HS, high salt; Veh, vehicle; AS, atrasentan; EL, enalapril; HW, heart weight; BW, body weight; LuW, lung weight; TL, tibia length.

Among HS diet animals, all treated animal groups except for low dose atrasentan showed substantial attenuation of cardiac hypertrophy compared to the vehicle treated group (Table 1). The most benefit was seen in high dose atrasentan treated group. Enalapril treated group only showed a modest benefit. However, when combined with moderate dose of atrasentan, there was a significant and further decrease in cardiac hypertrophy and cardiac dysfunction suggesting an additive benefit of atrasentan in setting of RAS inhibition.

**Fig 2:**
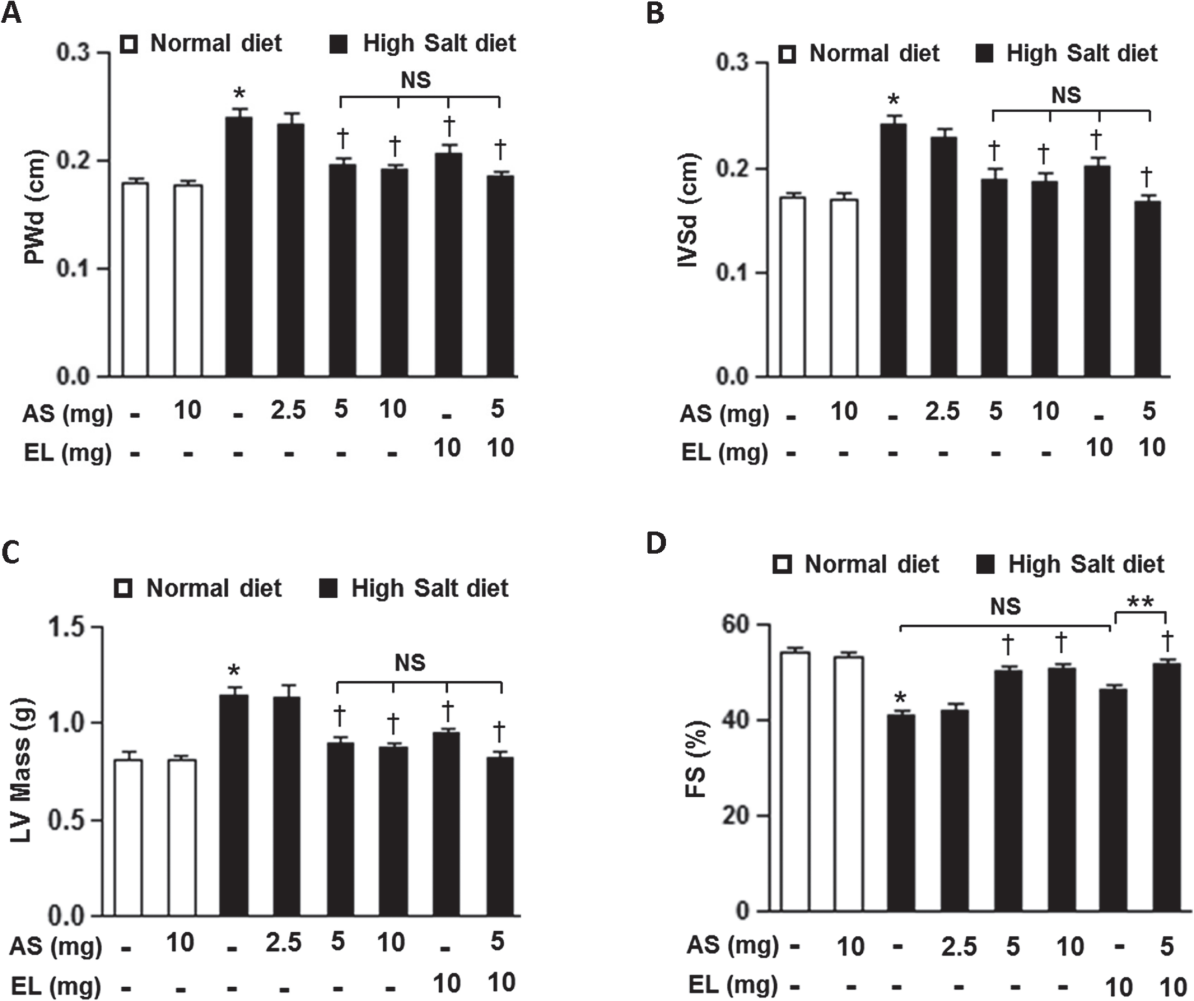
Echocardiographic finding in the DSS rats. *A-D*. Posterior wall thickness in diastole (PWd)(*A*), intraventricular septal wall thickness in diastole (IVSd)(*B*), left ventricular (LV) mass (*C*), and fractional shortening (FS)(*D*) of DSS rats from different groups. The results are expressed as means ± SEM. *n* = 5–8/group. *P < 0.05 vs. ND+V group; ^†^ *P* < 0.05 vs. HS+V group; **P < 0.05 vs. HS+EL group. NS, not significant; AS, atrasentan; EL, enalapril.

We further examined cardiac function with M-mode echocardiograms to evaluate the effectiveness of atrasentan in attenuation of cardiac hypertrophy and cardiac dysfunction. In HS+V rats, intraventricular septum thickness in diastole (IVSd), posterior wall thickness in diastole (PWd) of the left ventricle (LV) and LV mass were significantly increased (Fig. 2A-C) with significant decrease in fractional shortening (FS) as to compared to their ND littermates (Fig. 2D). Again, low dose atrasentan did not significantly affect the cardiac hypertrophy and cardiac dysfunction parameters in HS diet treated group. However, these cardiac parameters were all significantly improved in other treatment groups with the least benefit observed in enalapril only treated group. Of note, the group treated with combined therapy showed an additional beneficial effect and significantly increased these cardiac parameters over the enalapril treatment alone group.

### Atrasentan attenuates the fetal gene expression in the development of cardiac hypertrophy

**Fig 3:**
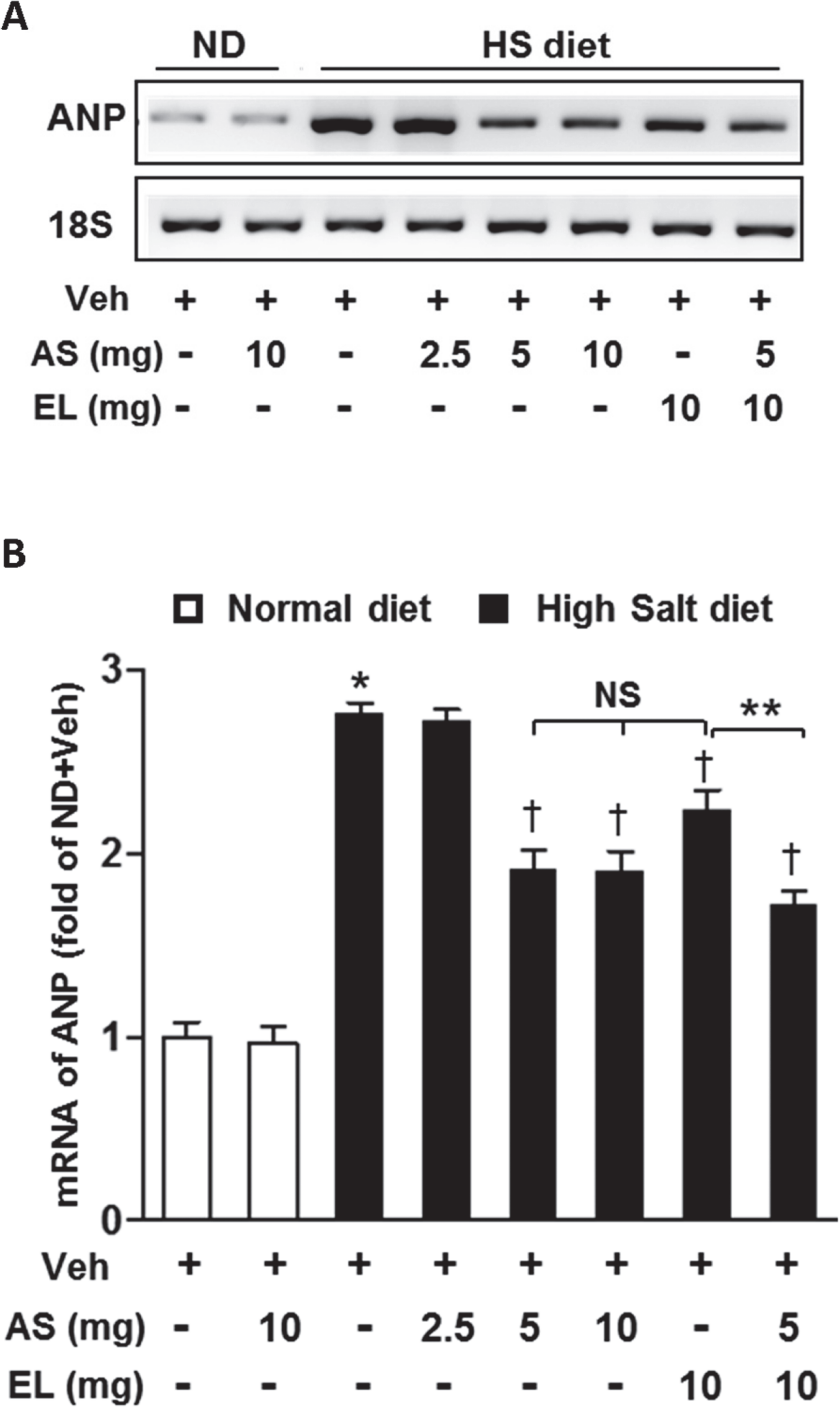
Effect of atrasentan on ANP mRNA expression *in vivo*. *A*. Representative mRNA expression of ANP in LV tissue. *B*. Quantitative analysis of mRNA expression of ANP. 18S expression is shown for internal loading control. The results are expressed as means ± SEM. *n* = 5–8 in each group. *P < 0.05 vs. ND+V group; ^†^ *P* < 0.05 vs. HS+V group; **P < 0.05 vs. HS+EL group. NS, not significant; Veh, vehicle; AS, atrasentan; EL, enalapril; HS, high salt; ND, normal diet.

Atrial natriuretic peptide (ANP) is a well-established biochemical marker of cardiac hypertrophy and heart failure. For further confirmation, we examined the mRNA expression level of ANP in LV tissues. Ventricular ANP mRNA levels were significantly elevated in HS+V group than those in ND animals (Fig. 3A-B). These increases were significantly attenuated by all treatment groups, except for low dose atrasentan group. Enalapril treatment alone showed the minimal attenuation of HS-diet induced ANP activation. In groups treated with combination of a moderate dose atrasentan and low dose enalapril, there was an additive effect in further decreasing ANP compared with the group treated with enalapril alone.

### Effects of atrasentan on renal impairment

**Fig 4:**
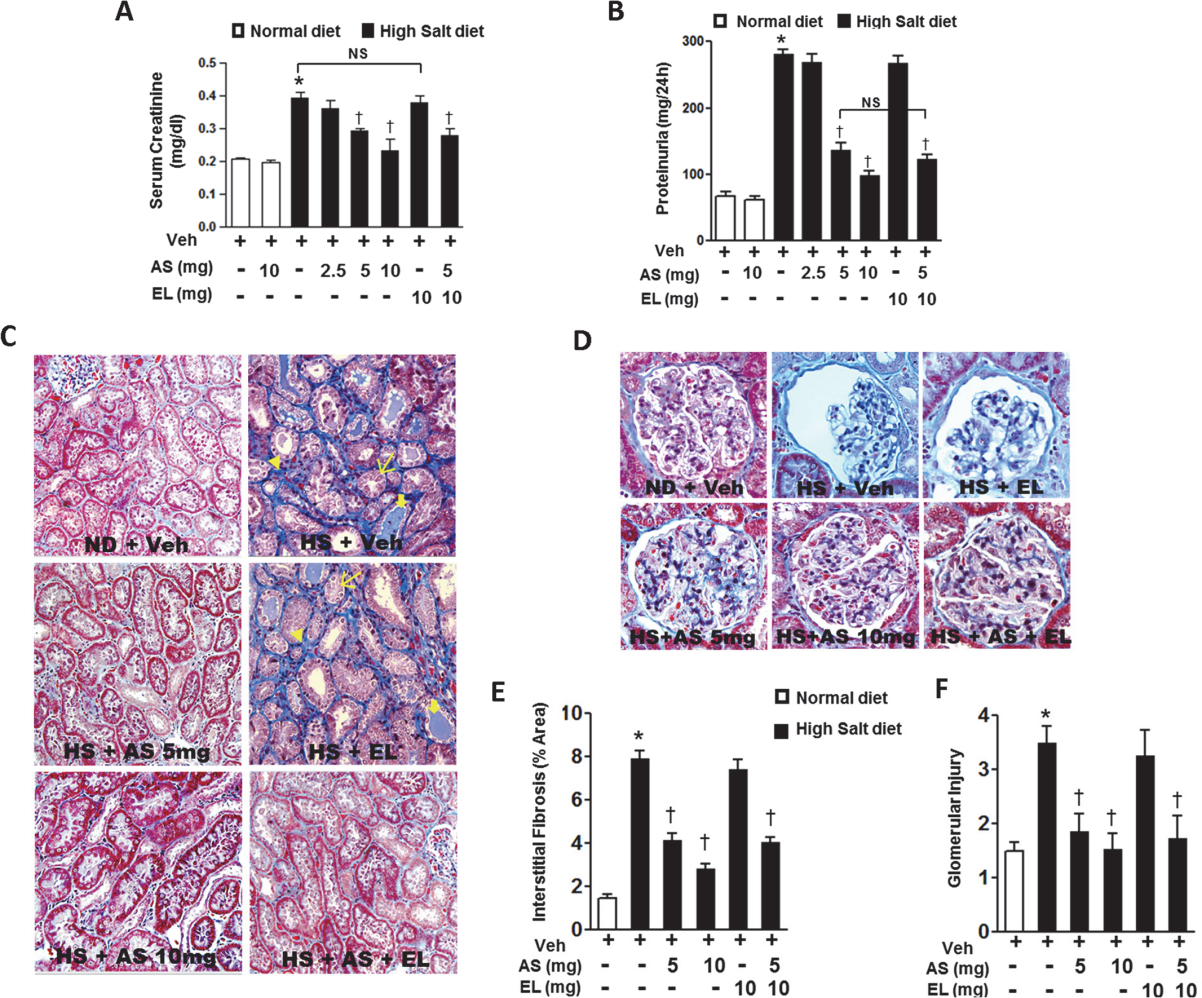
Effect of atrasentan on kidney function in DSS rats. *A-B*. Serum creatinine levels (*A*) and urinary protein levels (*B*) in DSS rats from different groups. The results are expressed as means ± SEM. *n* = 5–8/group. *P < 0.05 vs. ND+V group; ^†^ *P* < 0.05 vs. HS+V group. *C-D*. Representative photomicrographs of renal morphology (*C*) and glomerular injury (*D*) from: Dahl rats on normal diet with vehicle; high salt with vehicle, high salt diet with enalapril, high salt diet with moderate dose atrasentan, high salt diet with high dose atrasentan, and high salt diet with combined therapy (MT). *n* = 5–8 animals/group. *E-F*. Morphometric analysis of tubulointerstitial fibrosis (*E*) and glomerular injury (*F*). The results are expressed as means ± SEM. 20 random images per animal, *n* = 5–8 animals/group *P < 0.05 vs. ND+V group; ^†^ *P* < 0.05 vs. HS+V group. Veh, vehicle; EL, enalapril; ND, normal diet; HS, high salt; AS, atrasentan.

Salt-loaded DSS rats developed severe hypertension, proteinuria, glomerulosclerosis, and elevation of serum creatinine level in 6 weeks of HS diet [31]. In our study, there were significant differences in serum creatinine and proteinuria between ND and HS diet rats with vehicle (Fig. 4A-B), indicating marked hypertension associated with proteinuria and renal glomerular damage after 6 weeks of HS diet. The monotherapy with low dose atrasentan or enalapril did not significantly improve renal dysfunction and proteinuria in HS diet groups. However, moderate or high of atrasentan, and the combined therapy with atrasentan and enalapril significantly attenuated the elevated serum creatinine and proteinuria compared with the HS+V treated littermates.

Morphological analysis of kidney sections of DSS rats fed ND demonstrated normal glomeruli and interstitium (Fig. 4C-D, top left panel). However, high salt diet induced interstitial fibrosis (arrowhead), dilatation of tubules with tubular atrophy (thin arrows) and presence of intratubular cast (thick arrows) (Fig. 4C, top right panel) and glomerular necrosis (Fig. 4D, top middle panel). Expansion of the interstitium, indicated by an increase in the distance between the tubules, was also evident. These findings suggest that the DSS rats on HS diet with vehicle developed global glomerular sclerosis, interstitial fibrosis, atrophy, and dilated tubules. In DSS rats fed HS diet and treated with moderate & high dose atrasentan as well as combined therapy, these pathological changes were attenuated with relatively normal appearing glomeruli and interstitium (Fig. 4C, middle left, lower left and right panel, and Fig. 4D, lower panel). No improvement of renal pathology was observed in monotherapy with enalapril compared with the HS+V treated littermates (Fig. 4C, middle right panel and Fig. 4D, top right panel). The extent of tubular injury and degree of interstitial fibrosis and glomerular injury were significantly reduced in the moderate and high dose atrasentan monotherapy groups and the combined therapy group, compared with the vehicle treated rats with HS diet (Fig. 4E-F).

### Effects of atrasentan on hemodynamics

**Fig 5:**
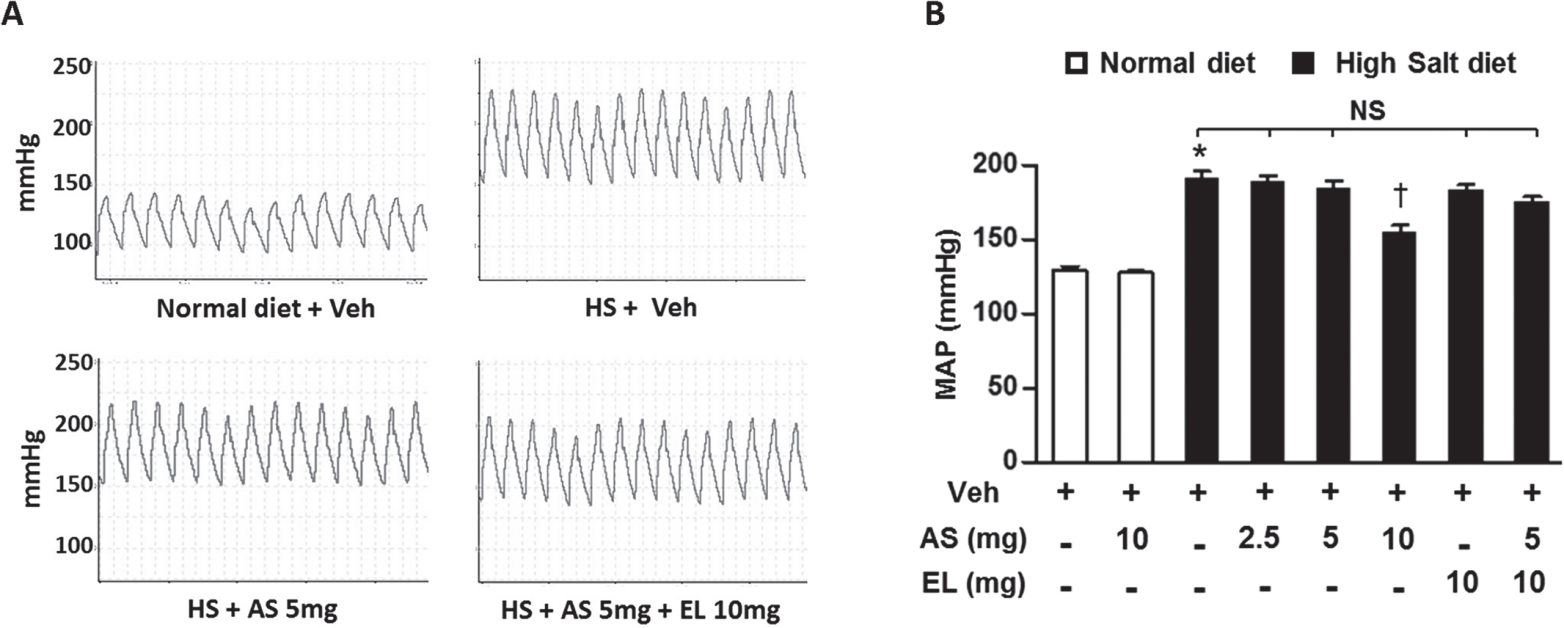
Hemodynamic finding in the DSS rats. *A*. Representative blood pressure tracings of DSS rats from different groups, *B*. Quantitative analysis of mean arterial pressure (MAP) in DSS rats from different groups. The results are expressed as means ± SEM. *n* = 5–8/group. *P < 0.05 vs. ND+V group; ^†^ *P* < 0.05 vs. HS+V group. NS, not significant; Veh, vehicle; AS, atrasentan; EL, enalapril; HS, high salt.

Excess salt intake is known to elevated mean arterial pressure (MAP) of DSS rats fed a HS diet compared to the rats fed a ND diet [23]. In our experiment, there was a 48% increase in MAP in HS+V group compared with ND diet group (Fig. 5A-B). In HS treatment groups, treatment with high dose atrasentan significantly decreased MAP when compared with their HS vehicle littermates. However, MAPs of other treatment groups were non-significant compared to the HS+V treated group. These data provide further evidence that high dose atrasentan significantly reduce BP, whereas moderate dose atrasentan or low dose enalapril alone or in combination do not significantly reduce BP. These findings suggest that the improvement in cardiac and renal dysfunctions by atrasentan—and to more modest extent in enalapril—is independent of lowering BP.

## Discussion

In this study with DSS rat model, we found that moderate and high dose of atrasentan had a substantial preventive effect on the progression of cardiac hypertrophy. Moreover, both atrasentan and enalapril, at a dose that does not alter BP, attenuated the clinical and biochemical evidence of heart failure. In fact, there was an additive anti-hypertrophic effect in combination therapy with atrasentan and enalapril suggesting a potential RAS-independent mechanism by atrasentan. Our study also revealed that moderate dose of atrasentan normalized serum creatinine level and restored normal renal architecture of glomeruli and interstitium without changing BP. However, enalapril, at a dose that does not alter BP, did not evoke any therapeutic effect on renal injury in hypertensive rats. The present study confirmed our hypothesis that in salt induced hypertension in DSS rats, the cardio-renal protective effect of atrasentan, an ET_A_ receptor antagonist, is independent of BP and RAS.

Endothelin, an endothelial cell-derived potent vasoconstrictor, has 4 isoforms (ET-1 through 4)[19,32]. Among the 4 ETs, ET-1 plays a major role in the cardiovascular system [32,33]. It regulates vascular tone, exerts positive inotropic and chronotropic effects as well as causes cardiac hypertrophy [34,35] by binding to specific membrane receptors, ET_A_ and ET_B_ [36]. ET-1 expression level is elevated under certain pathological conditions such as heart failure, renal failure and salt sensitive hypertension [37–40]. ET_A_ receptors are abundantly expressed in vascular smooth muscle cells and cardiomyocytes [41], and mediate the ET-1 concentration-dependent vasoconstriction and mitogenic actions of ET-1. However, ET activating ET_B_ receptors located on the vascular endothelium causes vasodilation through the production of nitric oxide and prostacyclin [42,43]. Theoretically, selective ET_A_ receptor antagonists should be more effective than nonselective ET_A_/ET_B_ receptor antagonists, given the role played by ET_B_ receptors in both vasodilation and ET-1 clearance [42].

Several studies also showed that ET-1 receptor antagonist prevented endothelial dysfunction, blocked renal damage, attenuated cardiovascular remodeling and heart failure, and improved survival rate [44,45]. In 2002, on the basis of convincing evidence of improved clinical status and survival in patients, US Food and Drug administration approved orally active ET receptor antagonist, bosentan (Tracleer), for the treatment of pulmonary arterial hypertension (PAH) [42]. Since then, several ET receptor antagonists have become available for the treatment of PAH/CKD including ambrisentan (Letairis, Volibris), sitaxsentan (Thelin), Avosentan, BQ123 and atrasentan [42,46]. Recently, Phase 3 clinical study called SONAR (Study Of Diabetic Nephropathy with Atrasentan) is ongoing to assess the effects of atrasentan—when added to standard of care—on progression of kidney disease in patients with stage 2 to 4 CKD and type 2 diabetes [47]. Atrasentan has been shown to be a potent and highly selective ET_A_ receptor antagonist [25,48]. In this study, we demonstrated that atrasentan, a selective ET_A_ receptor antagonist, alone has a significant anti-hypertrophic and cardioprotective effect even at a moderate dose without reducing BP. Interestingly, the high dose did not exert additional impact on cardiac hypertrophy and cardiac function compared with the moderate dose. It can be postulated that the higher dosages of the ET_A_ receptor antagonists may have resulted in an additional blockade of the ET_B_ receptors. Nonetheless, atrasentan has been shown to have 1000-fold greater affinity to human ET_A_ receptors than ET_B_ receptors [25].

RAS activation stimulates cardiomyocyte hypertrophy and fibroblast proliferation, thereby leading to left ventricular hypertrophy (LVH) and heart failure [49,50]. ACEi reduces the progression of RAS induced cardiac remodeling [51]. Enalapril maleate, one of the most commonly used ACEi [50], is non-hypertensive at a dose 10 mg/kg/day in spontaneously hypertensive rats (SHR)[52] and at least four times less potent in DSS rats than in spontaneous hypertensive rats (SHR) [53]. Our current study showed that enalapril dose that does not alter BP modestly limited LV remodeling without changing MAP. In comparison, the concomitant administration of atrasentan and enalapril, at a non-BP altering dose, considerably improved cardiac function and attenuated ANP expression level over enalapril monotherapy. Nonetheless, it did not illustrate any additional improvement of LV function and cardiac remodeling compared with atrasentan alone. In this context, the effect of ET blockade might be more important for cardiac and renal protection.

It is known that ET-1 induces cardiac hypertrophy through G-protein coupled receptors in cardiomyocytes and stimulates myocyte growth and myofibrillogenesis. ET-1 mainly uses the mitogenactivated protein kinase and phosphatidylinositol-3 kinase/AKT pathways to activate GSK3β, and the extracellular signal-regulated kinase 1/2 (ERK1/2) cascade [54,55]. In a healthy condition, ET-1 binding to ET_A_ promotes vasoconstriction, cell proliferation, collagen synthesis, and matrix accumulation [46,56,57], whereas ET_B_ activation pertains to vasodilation, anti-proliferation, and anti-fibrosis [46,58]. Selective ET_A_ receptor blocker has been shown to decrease LV interstitial collagen deposition [58,59], fibrosis and matrix metalloproteinase activity [60,61] in hypertensive rats. ET_B_ mediates vaso-relaxation by increasing the formation of prostacyclin and nitric oxide (NO) as well as the clearance of circulating ET-1[62]. In the case of highly selective ET_A_ receptor antagonist, ET_B_ remains active and therefore it can be regarded as “endogenous antagonistic receptors” which counteracts the effects of the ET_A_ receptor [63]. Altogether, these may result in an increased cardiac output and may prevent deterioration of global LV function.

Growing evidence suggests that RAS and ET-1 [64,65] has an important role in the development and progression of CKD. In the past decade, several experimental and clinical trials have suggested that ET receptor antagonist alone and/or combination with a blocker of the RAS has unique nephroprotective effects with a reduction of up to 45% in albumin [22,46,66–68]. Proteinuria is a common feature of CKD [14] and has an important contribution to CVD risk in CKD [69]. ACEi and angiotensin receptor blockers are standard therapy to prevent CKD. Phase 2 clinical study indicates that low doses of atrasentan substantially reduce urinary albumin excretion in diabetic patients with minimal concern for fluid retention [22]. Though, fluid retention occurrence with atrasentan is dose dependent and associated with only the short-term administration, since no statistically significant edema was observed after a long-term administration [43,70]. However, whether improvement in proteinuria correlates with cardiovascular outcome, and whether the beneficial effects of atrasentan is independent of RAS inhibition is not yet known. In this study, after 6 weeks, DSS rat on high salt diet developed severe hypertension, proteinuria, and glomerulosclerosis [32,71–73]. Monotherapy of enalapril, at a dose that didn’t modify blood pressure, did not have a significant effect on salt-induced proteinuria and serum creatinine. This result concurs with the previous study which showed renoprotective effects of ACEi are BP dependent [74]. Conversely, atrasentan, even at a dose that didn’t alter BP, significantly decreased serum creatinine and proteinuria, and mitigated glomerular sclerosis, interstitial fibrosis, atrophy and dilatation of tubules, and tubular casts in salt-loaded DSS rats. As stated above, earlier studies suggest that chronic blockade of ET_A_ reduces proteinuria and renal inflammation in various rat models without significant change in arterial pressure [15,64]. ET_A_ antagonism induces regression of glomerulosclerosis and albuminuria independent of hypertension and CKD regression associated with the improvement of renal blood flow and cortical microvasculature structure in the stenotic kidney [46,75,76]. However, the combined therapy of non-antihypertensive dose of atrasentan and enalapril did exert additional effect over monotherapy of atrasentan.

Our present findings indicate that BP independent monotherapy of ET_A_ receptor antagonist attenuated the progression of CKD and significantly mitigated CVD. This evidence proposes that proteinuria is strongly correlated with cardiovascular abnormalities in salt-loaded hypertensive rats, and that attenuation of proteinuria was indicative of reno- and cardioprotective effects. In BP independent combined therapy, main therapeutic index was exerted by ET_A_ receptor antagonist and was independent of RAS inhibition. All these findings suggest that, in patient with CKD and CVD, BP independent monotherapy of ET_A_ receptor antagonism may be considered a novel strategy.

## Supporting Information

S1 Methods This is the S1 word file for the supplemental detailed materials and methods. (DOCX)
