# Supplementary material for: Endothelin A Receptor Antagonist, Atrasentan, Attenuates Renal and Cardiac Dysfunction in Dahl Salt-Hypertensive Rats in a Blood Pressure Independent Manner
Source: PLoS One. 2015 Mar 16;10(3):e0121664. doi: 10.1371/journal.pone.0121664 (PMC4361570; doi:10.1371/journal.pone.0121664)
Supplement: S1 Methods — (DOCX) [file pone.0121664.s001.docx]

**Supplementary Information**

**Endothelin A receptor antagonist, atrasentan, attenuates renal and cardiac dysfunction in Dahl salt-hypertensive rats in a blood pressure independent manner**

Mohammed A. Samad^1, 2^, Ui Kyoung Kim^1^, Joshua J. Kang^1^, Qingen Ke^1^, Peter M. Kang^1, 2^

**Materials and Methods**

**Adult cardiomyocyte culture**

Primary cultures of cardiomyocytes from 6-week-old Sprague-Dawley rats. The hearts were removed rapidly using sterile technique, attached to the Langendorff apparatus and perfused retrogradely for 5 minutes with calcium-free perfusion buffer containing minimum essential medium (Joklik’s modification) supplemented with 5 mM taurine, 2 mM creatine, 5 mM HEPES, and 20 u/L insulin. The hearts were then enzymatically dissociated with perfusion buffer containing 0.3% collagenase for 45 minutes, then minced further and dissociated in incubation buffer (perfusion buffer with 0.2% BSA and 0.3 mM CaCl2) containing 0.3% collagenase. The supernatants containing dissociated cardiomyocytes were washed twice with incubation buffer. Myocyte fractions were separated and plated on laminin coated plates (10 μg/ml) at 2x10^5^ cells/cm^2^ with serum-free DMEM supplemented with 5 mM taurine, 5 mM creatine, 2 mM L-carnitine, 25 mM HEPES and 20u/L insulin. After an hour of plating, unattached (damaged or dead) cells were removed by changing the media. The cells were plated for 24 hours. Preparation of each culture required the use of a different heart.

**Semi-quantitative RT-PCR**

We used following primers to measure ANF and 18S mRNA expression.

| **Name of primer** | **Sequence** | **Product size (bp)** | **Reference** |
| --- | --- | --- | --- |
| ANP | sense- 5′-GCCGGTAGAAGATGAGGTCA-3′  antisense- 5′-GGGCTCCAATCCTGTCAATC-3′ | 269 | (29) |
| 18S | sense- 5′-GTTATGGTTCCTTTGTCGCTCGCTC-3′ antisense- 5′-TCGGCCCGAGGTTATCTAGAGTCAC-3′ | 209 | (24) |

**Pathological analysis**

Rats were weighed and then anesthesized. The chest cavity was rapidly opened and blood was collected and left on ice for 20 minutes to allow clotting. Serum was separated by centrifuging at 1,500x g for 10 minutes and creatinine levels were measured by using IDEXX Catalyst^TM^ Test kit for creatinine (IDEXX, ME, USA) immediately. Heart was removed and rinsed twice with ice cold saline. Major blood vessels and connective tissue were removed, the heart blotted dry, and the heart weight (HW), lung weight (LuW), liver weight (LiW) and tibial length (TL) were measured. Hearts were stored at -80^o^C and kidneys were fixed in 10% formalin until further analysis.

**Echocardiography**

Transthoracic echocardiography was performed using Agilent Sonos 5500 sector scanner ultrasound machine equipped with a 7.5 MHz transducer at the end of study period on each group of animals. Rats were anesthetized with ketamine and xylazine mixture and 2D-guided M-mode tracings were used to record anterior and posterior wall thickness and LV end systolic and end diastolic diameters dimensions, LV thickness at diastole, LV mass index, fractional shortening (FS% = (LVED – LVES/LVED)) were calculated. LV mass was calculated by using the corrected American Society of Echocardiography (ASE) simplified cubed equation: LV mass (grams) = 0.8 [1.05 (LVDd+AWd+PWd)^3^ − (LVDd)^3^].
